# Identification of a non-exported Plasmepsin V substrate that functions in the parasitophorous vacuole of malaria parasites

**DOI:** 10.1128/mbio.01223-23

**Published:** 2023-12-11

**Authors:** Aline Fréville, Margarida Ressurreição, Christiaan van Ooij

**Affiliations:** 1 Department of Infection Biology, London School of Hygiene & Tropical Medicine, London, United Kingdom; Stanford University, Stanford, California, USA

**Keywords:** malaria, proteolysis, *Plasmodium*, protein export, host–parasite relationship

## Abstract

Malaria parasites alter multiple properties of the host erythrocyte by exporting proteins into the host cell. Many exported proteins contain a five-amino acid motif called the *Plasmodium* export element (PEXEL) that is cleaved by the parasite protease Plasmepsin V (PM V). The presence of a PEXEL is considered a signature of protein export and has been used to identify a large number of exported proteins. The export of proteins becomes essential midway through the intraerythrocytic cycle—preventing protein export blocks parasite development 18–24 h after invasion. However, a genetic investigation revealed that the absence of the PEXEL protein PFA0210c (PF3D7_0104200) causes parasite development to arrest immediately after invasion. We now show that this protein is cleaved by PM V but not exported into the host erythrocyte and instead functions in the parasitophorous vacuole; hence, the protein was renamed PV6. We additionally show that the lysine residue that becomes the N-terminus of PV6 after processing by PM V prevents export. This is the first example of a native *Plasmodium falciparum* PM V substrate that remains in the parasitophorous vacuole. We also provide evidence suggesting that the parasite may produce at least one additional essential, non-exported PM V substrate. Therefore, the presence of a PEXEL and, hence, processing of a protein by PM V do not always target a protein for export, and PM V likely has a broader function in parasite growth beyond processing exported proteins. Furthermore, we utilized this finding to investigate possible requirements for protein export further.

**IMPORTANCE:**

In the manuscript, the authors investigate the role of the protease Plasmepsin V in the parasite–host interaction. Whereas processing by Plasmepsin V was previously thought to target a protein for export into the host cell, the authors now show that there are proteins cleaved by this protease that are not exported but instead function at the host–parasite interface. This changes the view of this protease, which turns out to have a much broader role than anticipated. The result shows that the protease may have a function much more similar to that of related organisms. The authors also investigate the requirements for protein export by analyzing exported and non-exported proteins and find commonalities between the proteins of each set that further our understanding of the requirements for protein export.

## INTRODUCTION

Export of proteins from malaria parasites into the host erythrocyte is an essential aspect of the parasite–host interaction—exported proteins induce changes in the permeability of the host cell, adhesiveness to the endothelium, and deformability of the cell (1, 2). Exported proteins are synthesized in the parasite and transported through the endoplasmic reticulum (ER) and secretory pathway, released into the parasitophorous vacuole, and subsequently transported past the parasitophorous vacuole membrane (PVM) through a large protein complex, the Parasite Translocon of EXported proteins (PTEX), into the host erythrocyte cytosol (3–7). The PTEX consists of three essential core subunits, HSP101, PTEX150, and EXP2, with several accessory proteins, of which PTEX88 and Trx2 are the most prominent (4, 7–10). An important step in our understanding of protein export was the discovery of a motif shared by many exported proteins, the *Plasmodium* export element (PEXEL; also referred to as host targeting motif) (11, 12). The PEXEL is a five-amino acid motif with the sequence RxLxE/D/Q [a K is sometimes allowed at the first position (13)]. The discovery of the PEXEL allowed the prediction of the set of exported proteins produced by malaria parasites, which revealed that these parasites encode hundreds of exported proteins (11, 12, 14, 15). The PEXEL is cleaved in the ER by the parasite protease Plasmepsin V (PM V) (16–20) during ER translocation (18, 21, 22). It has been speculated that cleavage by PM V designates proteins for export (16, 17), and to date, no substrates of PM V other than exported proteins have been identified. However, the inhibition of PM V during the schizont stage leads to a different phenotype than the inhibition of protein export—when PM V is inhibited starting at an early schizont stage, parasite development arrests immediately after the invasion of the parasite (23), in contrast to the phenotype of inhibition of the PTEX during the schizont stage, which leads to an arrest late in the ring stage (3, 5, 24, 25). This indicates that PM V may perform functions in addition to preparing proteins for export.

In addition to the PEXEL proteins, malaria parasites encode a second type of exported protein: the PEXEL-negative exported proteins (PNEPs) (26–28). Soluble PNEPs may be targeted to the ER by canonical N-terminal signal sequences (SSs), but many PNEPs lack a signal sequence and instead contain an internal hydrophobic sequence that targets the protein to the ER. After transport through the secretory pathway and delivery to the PV, PNEPs are also translocated through the PTEX (3).

Biochemical and structural investigations of the PTEX have provided important insight into the mechanism of protein transport (6, 7). Proteins require unfolding (29–31), mediated by HSP101 (7, 32), which also provides the energy for the translocation of the peptide chain through the pore of the PTEX complex, which is formed by EXP2 (7, 33). Exactly how parasite proteins are recognized by the PTEX has remained unclear. It has been speculated that processing by PM V acts as a signal that targets the protein for export (17, 34) and that exported proteins are potentially already recognized by HSP101 in the ER (34). Several studies have indicated that an important factor that determines export is the nature of the N-terminal residue of the protein (31, 35). The processing of the protein by PM V in proteins that contain a PEXEL, which occurs between the L and the second x residue in the PEXEL (16, 17), produces a protein in which x becomes the new N-terminal residue; changing the protease that processes model exported proteins had no effect on the export of the protein if the new N-terminal residue in the processed protein remained the same (35). However, these experiments were performed using native exported proteins. In this study, we focused on the *Plasmodium falciparum* phospholipid transfer protein PFA0210c (PF3D7_0104200) previously identified as essential for early parasite development (36, 37). This protein is part of a small subset of PEXEL proteins that are conserved among all *Plasmodium* species and can mediate the transfer of a wide range of phospholipids between membranes (37, 38). Here, we show genetically, biochemically, and microscopically that PFA0210c is a native *Plasmodium falciparum* PM V substrate that is not exported but instead functions in the PV and hence propose to rename it PV6. We also provide evidence that the parasite likely produces at least one other PM V substrate that is not exported. Our findings show that the processing of substrates by PM V does not *per se* target a protein for export and that the protease has an additional role in processing parasite proteins important for the development of the parasite immediately after invasion. Furthermore, we use our previous findings to analyze the sequences of exported and non-exported proteins to refine the signal for protein export further.

## MATERIALS AND METHODS

### Parasites

All experiments were performed with *P. falciparum* strain 3D7 and the transfected parasite line PfBLD529 (PV6-diCre) containing the floxed *pv6* (*pfa0210c/PF3D7_0104200*) gene described previously (36). Parasites were maintained as described previously (39) in RPMI-1640 medium (Life Technologies) supplemented with 2.3 g/L sodium bicarbonate, 4 g/L dextrose, 5.957 g/L HEPES, 0.5% AlbuMax II, 50 µM hypoxanthine, and 2 mM L-glutamine (cRPMI). Cultures were maintained at 3% hematocrit and incubated at 37°C. Human erythrocytes were obtained from the National Blood Transfusion Service, UK, and Cambridge Bioscience, UK.

Parasites were synchronized by isolating schizonts on a Percoll cushion and allowing these parasites to invade uninfected erythrocytes for 1–3 h. The remaining schizonts were removed on a second Percoll cushion. Rapamycin treatment was performed as described previously (40, 41). Briefly, rapamycin was added to parasite cultures approximately 30 h post-invasion to a final concentration of 10 nM (from a stock solution of 100 µM in DMSO), and cultures were incubated at 37°C for 1 h. Parasites were then pelleted and resuspended in cRPMI. Control cultures were treated similarly using DMSO instead of rapamycin.

Parasites were transfected using the schizont transfection protocol (42). Briefly, parasites were synchronized using Percoll cushion purification to set up the invasion of fresh erythrocytes for 1–3 h followed by sorbitol lysis of remaining schizonts. To prepare parasites for transfection, schizonts were purified on a Percoll cushion and allowed to invade fresh erythrocytes. After 1–3 h, the remaining schizonts were removed from the culture on a Percoll cushion and transferred to a 15 mL tube. These schizonts were washed with cRPMI, transferred to microcentrifuge tubes, and pelleted. The schizonts were subsequently resuspended in a 100 µL AMAXA nucleofection P3 Primary Cell transfection reagent and 10 µL TE containing 15–30 µg targeting plasmid and 20 µg plasmid pDC2-Cas9-hDHFRyFCU that encodes the appropriate guide RNA and Cas9 (41), followed by transfection using the nucleofection device (AMAXA), program FP 158. Transfected schizonts were mixed with uninfected erythrocytes and incubated with shaking at 37°C for 30 minutes to allow invasion. Drug selection using 2.5 nM WR99210 was initiated after 24 h and maintained for 7 days. Transfected parasites were, in most cases, recovered after 3–4 weeks.

### Plasmids

Plasmid pBLD634 encoding full-length PV6 with a K64A mutation, controlled by the native promoter, was made as follows. First, the K-A mutation was introduced using overlapping PCR. The 5′ and 3′ regions were amplified using primer pairs CVO081-CVO520 and CVO519-CVO023 (see Table S1 for primer sequences), respectively, using *P. falciparum* 3D7 gDNA and a PV6-HA_3_-His_6_-GFP fusion (37) as templates. The resulting fragments were cloned into pBLD619 (this plasmid was made by cloning 1,938 bp upstream of *pv6* into pBLD466 [also described as pBSPfs47DiCre (41)], which contains two homology regions for the integration of the plasmid into the *pfs47* locus (41) to produce pBLD634. These plasmids were integrated into the *pfs47* locus as described, using plasmid pDC287, which encodes Cas9 and a guide RNA targeting *pfs47*, as described previously (41).

EXP1-HA_3_-PV6, AMA1-SS-HA_3_-PV6, and PV6-HA_3_-PV6 expression plasmids were made as follows. Sequences encoding the EXP1-SS-HA_3_-PV6 and the 5′ region of PV6 (spanning the fraction of the gene from the start ATG to the PEXEL) were synthesized as a gene fragment codon optimized for *Escherichia coli* (with further manual curation) to decrease the similarity with the native *exp1* and *pv6* genes. The fragments were cloned into pCR-Blunt (Invitrogen), producing pBLD701 and pBLD703, respectively. Subsequently, the 1,086 bp downstream of the *pv6* gene was amplified using primers CVO675 and CVO676 and introduced into pBLD701 at the AflII site using InFusion (Takara), producing pBLD702. The fragment containing EXP1-HA_3_-PV6 and sequence downstream of *pv6* was released and cloned into pBLD619, which contains two homology regions for integration of the plasmid into the *pfs47* locus (41) at the AvrII and AflII sites, producing pBLD706. Subsequently, the region encoding the EXP1 signal sequence was replaced by the recodonized region encoding the PV6 N-terminus (from pBDL703) or the AMA1 N-terminus using AvrII and XhoI (amplified from gDNA using primers CVO714 and CVO730), yielding pBLD708 and pBLD728, respectively. pBLD708 and pBLD728 were integrated into the *pv6* locus using Cas9-mediated gene modification as described previously (36).

### Polymerase chain reaction

Verification of modification of the *pv6* locus was performed by first isolating genomic DNA from transfected parasites (Monarch genomic DNA isolation kit, New England Biolabs). The integration of targeting plasmids at the *pfs47* locus was determined with primer pairs CVO119-CVO120 (wild-type locus and integrated locus), CVO119-CVO070 (integration-specific), and CVO120-CVO694 (integration-specific). PCRs were set up according to the manufacturer’s instructions using Q5 Polymerase (New England Biolabs). The integration of targeting plasmids at the *pv6* locus was tested with the primer pairs CVO689-CVO111 (wild-type 5′ end), CVO071-CVO183 (wild-type 3′ end), CVO689-CVO586 (integrant 5′ end), and CVO321-CVO183 (integrant 3′ end) using Q5 Polymerase (New England Biolabs). PCRs were set up according to the manufacturer’s instructions using Q5 Polymerase (New England Biolabs) to amplify the 3′ end and CloneAmp (Takara Bio) for the amplification of the 5′ end.

### Microscopy

Images of Giemsa-stained smears were captured using an Olympus BX51 microscope equipped with a 100× oil objective and an Olympus SC30 camera controlled by cellSens software.

**Fig 1:**
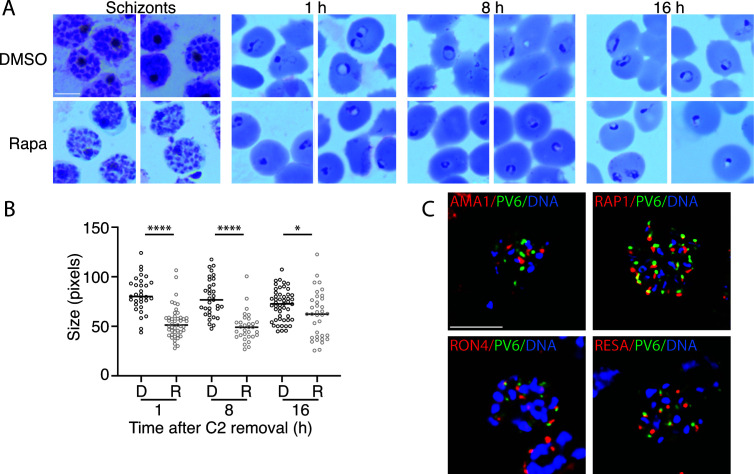
Development of parasites expressing or lacking PV6 during the ring stage and PV6 localization in schizonts. (**A**) Synchronized parasites with a floxed *pv6* (*pfa0210c/ PF3D7_0104200*) gene were treated at the late trophozoite stage with DMSO or with rapamycin to induce the excision of *pv6*. Parasites were examined at the schizont stage in Giemsa-stained smears, and subsequent egress was synchronized with ML10 treatment. Giemsa smears were produced 1, 8, and 16 h after Compound 2 (C2) removal. The scale bar represents 5 µm. (**B**) Quantitation of the size of the parasite diameter in the samples shown in panel A. Data are based on the measurement of at least 30 rings. The Mann–Whitney *U* test was performed for statistical analysis: *P < 0.05; ****P < 0.0001. D, DMSO-treated; R, rapamycin-treated. (**C**) Costaining of schizonts with antibodies against PV6 (green) and the apical organelle markers AMA1 (micronemes), RAP1 (rhoptry), RON4 (rhoptry neck), and RESA (dense granule) (all in red) by immunofluorescence microscopy. DNA was stained with Hoechst 33342 (blue). Individual channels are shown in Fig. S1B. The scale bar represents 5 µm.

**Fig 2:**
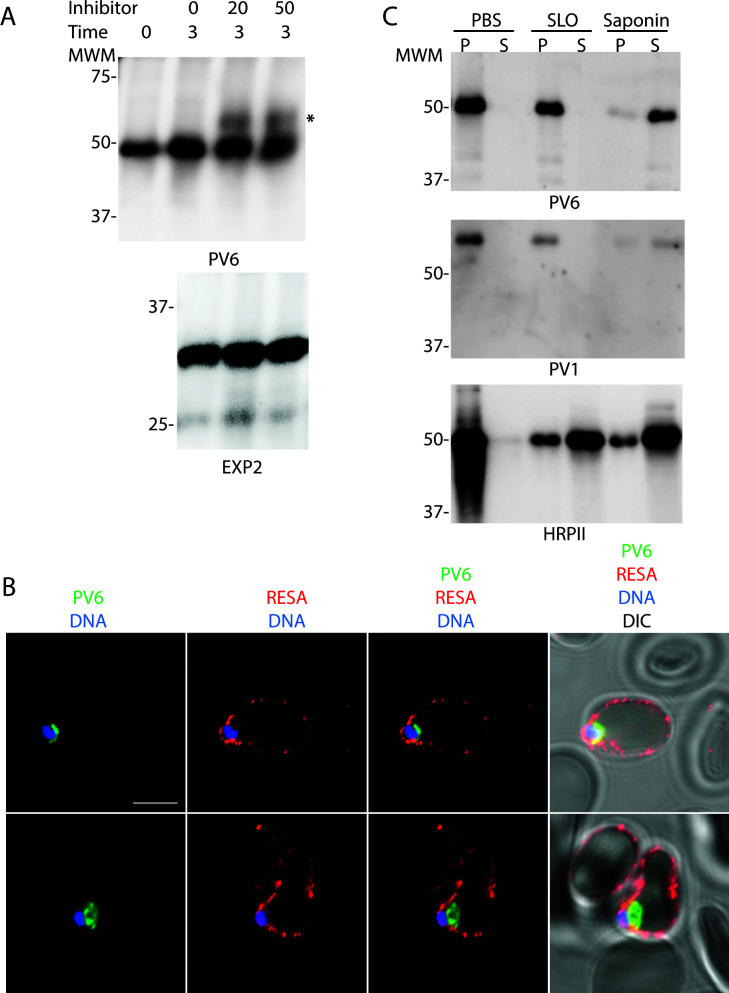
Role of Plasmepsin V in the processing of PV6 and localization of PV6 after invasion. (**A**) Parasites were treated with the indicated amount (in µM) of the PM V inhibitor WEHI-1252601 for 3 h. Parasite extracts were separated by SDS-PAGE and probed with antibodies against PV6 (top panel) and EXP2 (bottom panel). Note the presence of a higher molecular mass band in the inhibitor-treated samples in the anti-PV6 (indicated with *) but not the anti-EXP2 immunoblot. Expected sizes of unprocessed PV6 and PV6 processed by PM V: 53.6 and 46.4kD, respectively. (**B**) Localization of PV6 and RESA immediately after invasion of 3D7 parasites. Invasion was synchronized with ML10, and samples were collected within 2 h after the removal of ML10. Note that the erythrocyte at the top of the panel is also infected and therefore also displays anti-RESA staining but that the parasite in that cell is outside of the plane of focus (see Fig. S2A for a maximum projection of the image). The scale bar represents 5 µm. (**C**) Differential lysis of erythrocytes infected with trophozoite-stage parasites with streptolysin O (SLO) and saponin. Infected erythrocytes were treated with PBS, SLO, or saponin, the erythrocytes were pelleted, and the supernatant (S) and pellet (P) were collected. Samples were prepared for immunoblotting and probed with antibodies against the indicated proteins. Note the similarity in the release of PV6 and the parasitophorous vacuole marker PV1 in the samples.

**Fig 3:**
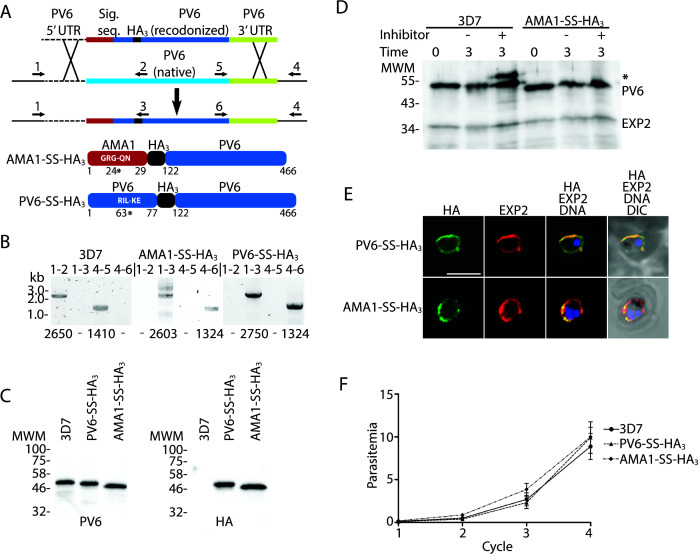
Replacement of native PV6 with an AMA1-SS-HA_3_-PV6 fusion. (**A**) Outline of the replacement strategy of the native *pv6* gene with the gene encoding the AMA1-SS-HA_3_-PV6 fusion. The same strategy was used to create the PV6-HA_3_-PV6 fusion. Cartoons at the bottom show the expected protein products after the replacement of the native gene with the inserted gene. Indicated in white letters is the predicted signal sequence cleavage site in the AMA1-SS-HA_3_-PV6 fusion and the PEXEL sequence of the PV6-HA_3_-PV6 fusion. The number and * indicate the signal sequence cleavage site in the AMA1-SS-HA_3_-PV6 and PM V cleavage site in the PV6-HA_3_-PV6 fusion. Numbers indicate amino acid residues of the native proteins. (**B**) Integration PCR using wild-type and transfected parasite DNA. Primer pairs are indicated above the lanes (see also Table S1), primer binding sites are indicated in panel A, and expected sizes of PCR products are indicated below each lane. Note the absence of integration-specific PCR products in the samples using gDNA from wild-type parasites and the absence of wild-type-specific PCR products in the samples using gDNA from transfectants. (**C**) Anti-PV6 (left) and anti-HA (right) immunoblots of wild-type (3D7) and transgenic parasite (PV6-HA_3_ and AMA1-SS-HA_3_) extracts using the indicated antibodies. Expected molecular weights are 46.4 kDa (3D7 native PV6), 45.4 kDa (PV6-HA_3_-PV6), and 44.4 kDa (AMA1-SS-HA_3_-PV6). (**D**) Effect of Plasmepsin V inhibitor treatment on PV6, AMA1-SS-HA_3_-PV6, and EXP2. The predicted sizes of uncleaved and cleaved proteins are 53.6 and 46.4 kDa (PV6), 47.3 and 44.4 kDa (AMA1-SS-HA_3_-PV6), and 33.4 and 30.8 kDa (EXP2). Note the absence of unprocessed AMA1-SS-HA_3_-PV6 and EXP2 in the presence of the Plasmepsin V inhibitor. * indicates the higher molecular mass product in the 3D7 lysate. (**E**) Localization of AMA1-SS-HA_3_-PV6 and PV6-HA_3_-PV6 fusion proteins. The fusion proteins were detected using an anti-HA antibody, and EXP2 was detected using an anti-EXP2 rabbit anti-serum. (**F**) Growth assay of 3D7, PV6-HA_3_-PV6, and AMA1-SS-HA_3_-PV6 parasites. Individual growth assays were set up in triplicate; the data shown are from three biological replicates. Error bars indicate the standard error of mean but are, in several cases, too small to protrude from the symbol.

For the colocalization of PV6 with apical organelle markers, late-stage parasites were purified on a Percoll gradient. The parasites were smeared on a microscope slide, air-dried, and stored at −20°C. Parasites were fixed with 4% paraformaldehyde in PBS, permeabilized with 0.1% Triton X-100, blocked with 3% BSA, and subsequently stained with antibodies against PV6 and an apical organelle marker [AMA1 (dilution 1:500), RAP1 (1:100), and Ron4 (1:500), all three kind gifts of Michael Blackman (Francis Crick Institute) and RESA (1:500), obtained from the Antibody Facility at The Walter and Eliza Hall Institute of Medical Research], followed by staining with fluorescently labeled secondary antibodies and staining of the DNA with Hoechst 33342 (5 µg/mL). To visualize PV6 after invasion, purified schizonts were allowed to invade fresh erythrocytes for approximately 1 h and subsequently fixed in solution with 4% paraformaldehyde and 0.01% glutaraldehyde in PBS for 1 h at room temperature (43). The parasites were permeabilized and stained as described above using anti-PV6 (36, 37) and mouse anti-RESA (Antibody Facility of the Biotechnology Centre of the Walter and Eliza Hall Institute of Medical Research) or mouse anti-EXP2 (European Malaria Reagent Repository) (1:2,000) antibodies and 5 µg/mL Hoechst 33342. The parasites were then stored at 4°C in a small amount of PBS (10–20 µL). For microscopy, 1.5 µL of resuspended parasites was placed on a polyethyleneimine-coated glass slide, mixed with 1.5 µL of Vectashield anti-fade mounting medium, and covered with a cover glass, which was then sealed with nail polish. The parasites shown in Fig. 1, 2, and 4 were imaged on a Nikon Ti-E inverted microscope with Hamamatsu ORCA-Flash 4.0 Camera and Piezo stage driven by NIS elements version 5.3 software. The images shown in Fig. 3 and Fig. S4D were acquired using a Zeiss LSM880 confocal microscope driven by Zen Black version 2.3 software. The emission wavelengths used were 462 nm (Hoechst), 548 nm (Alexa 488), and 637 nm (Alexa 568). The images were processed using FIJI. The resulting images were cropped and file sizes were altered using Photoshop, and figures were produced using Illustrator.

The export of PV6 K64A was visualized by fixing 3D7 parasites and parasites expressing PV6 K64A in solution and prepared for microscopy as described above for the visualization of PV6 after the invasion. The samples were stained with a mouse monoclonal anti-PV6 antibody (37) and rabbit anti-PV1 anti-serum (a kind gift of Eizo Takashima) or affinity-purified rabbit anti-PV6 (36) and mouse anti-EXP1 antibodies (a kind gift from Mike Blackman).

### Measurement of parasite size

To measure the size of parasites producing or lacking PV6 after invasion, PfBLD529 parasites were synchronized and treated with rapamycin or DMSO approximately 30 h after the initiation of invasion. Egress of the parasites was inhibited with the addition of compound 2 to 1.5 µM (42). To initiate a highly synchronized invasion, compound 2 was removed by washing the cells with cRPMI, after which samples were taken at the indicated time points. One hour after the removal of compound 2, it was added to the samples again to prevent further invasion. To prevent the repeated sampling from affecting parasite development, the DMSO-treated and rapamycin-treated cultures were each split into four small flasks, which were sampled sequentially. At the indicated time points, parasites were smeared and stained with Giemsa. The diameter of the parasite was determined by imaging the Giemsa-stained parasites using an Olympus BX51 microscope equipped with a 100× oil objective and an Olympus SC30 camera controlled by cellSens software. The size was determined by measuring the longest diameter in each parasite using FIJI/ImageJ software, using the line selection tool and set measurement function.

### Growth assays

To determine the parasite growth rates, parasites were synchronized using Percoll cushion as described above. Parasites containing a floxed version of the *pv6* gene were treated with rapamycin or DMSO as described above immediately after the invasion. The cultures were diluted to a parasitemia of 0.1%–0.5%, and growth was measured by removing a 50 µL aliquot and fixing that in an equal volume of 2× fixative and stain (8% paraformaldehyde, 0.2% glutaraldehyde, 2× SYBR Green). Fixed samples were stored at 4°C. To prepare the samples for cytometry, the fixative was removed, and the cells were resuspended in 1 mL PBS. A 200 µL aliquot was transferred to a 96-well plate, and the parasitemia was measured using an Attune cytometer outfitted with a CytKick autosampler (ThermoFisher). The laser settings used for the detection of the erythrocytes and parasites were as follows: forward scatter 125 V, side scatter 350 V, and blue laser (BL1) 530:30 280V. The experiment was set up in triplicate, with a minimum of three biological replicates.

### Streptolysin O and saponin treatment and immunoblots

SLO (SigmaAldrich) was titrated to quantify the hemolytic units per microliter. To perforate the erythrocyte plasma membrane to release erythrocyte cytosol proteins, 10^8^ erythrocytes infected with trophozoite-stage parasites (approximately 30 h post-invasion) were pelleted and resuspended in 100 µL PBS containing four hemolytic units SLO. To release erythrocyte cytosol and PV proteins, 10^8^ parasites were pelleted and resuspended in 100 µL 0.1% saponin in PBS. As a control, 10^8^ parasites were pelleted and resuspended in 100 µL PBS. The treated cells were pelleted; the supernatant of these samples was collected and mixed with an equal volume of 2× SDS-PAGE loading dye, and the pellet was resuspended in 1× SDS-PAGE loading dye.

### Immunoblots

Parasite lysates were separated by SDS-PAGE, transferred to nitrocellulose, and probed with anti-PV6 (1:1,000), anti-HRPII (Immunology Consultants Laboratory, 1:2,000), anti-aldolase-HRP (Abcam, 1:5,000), anti-PV1 (1:1,000), anti-EXP2 (1:2,000), or anti-HA (3F11, Merck; 1:2,000) antibodies. Proteins were visualized by incubating the blots with the appropriate HRP-linked anti-mouse or anti-rabbit secondary antibodies and developing them using Clarity ECL Western blotting substrates (Bio-Rad).

### Plasmepsin V inhibitor treatment

To determine whether PV6 is a PM V substrate, infected erythrocytes in a synchronized parasite culture were isolated on a Percoll gradient. Approximately 30 h after invasion, parasites were pelleted and either resuspended in 1× SDS-PAGE loading dye or resuspended in cRPMI containing 0, 20, or 50 µM PM V inhibitor WEHI-1252601 (a kind gift of Justin Boddey). These parasites were incubated at 37°C for 3 h and then pelleted and resuspended in 1× SDS-PAGE loading dye. Lysates were separated by SDS-PAGE, transferred to nitrocellulose, and probed with anti-PV6 (1:1,000) or anti-EXP2 (1:2,000) antibodies.

To determine the effect of PM V inhibition on invading parasites, cultures of 3D7 and AMA1-SS-HA_3_-PV6 parasites were synchronized, and half the culture was treated with 7.5 µM WEHI-1252601 approximately 40 h after invasion. Egress was blocked by the addition of ML10 to 25 nM (39, 44); when most parasites appeared to have arrested, the parasites were pelleted, and ML10 and WEHI-1252601 were removed. The culture that had been treated only with ML10 was split into two, and WEHI-1252601 was added to one of the two cultures. The parasites were incubated at 37°C to allow egress and invasion of fresh erythrocytes. Parasites were collected 1 h after the removal of ML10, smeared on slides, and stained with Giemsa.

To determine the effect of inhibition of PM V on parasites expressing or lacking PV6, similar experiments were carried out on PfBLD529 parasites. The parasites were synchronized and treated with rapamycin or DMSO approximately 30 h after initiation of invasion and allowed to develop to the schizont stage, at which point they were subjected to ML10 blocking and WEHI-1252601 treatment for 4 h at 37°C. After the removal of ML10 and WEHI-1252601, the parasites were allowed to egress and invade fresh erythrocytes for 1 h. The Giemsa-stained parasites were imaged, and the measurement of the parasite size was performed as described above.

## RESULTS

### A *pv6* mutant does not develop after invasion

In a previous investigation of a *pv6 loxP* mutant (PfBLD529) in which the native *pv6* gene can be removed by the addition of rapamycin in the parasite culture, we observed that its phenotype became apparent during the ring stage of the intraerythrocytic developmental cycle, very soon after the invasion of the parasite. Rapamycin-treated parasites formed dysmorphic rings with a translucent center that did not develop further (36). In contrast, mutants lacking subunits of the PTEX that are unable to export proteins develop seemingly as normal through the ring stage until the ring–trophozoite transition (18–24 h post-invasion), indicating that the functions of exported proteins are not essential until this stage (3, 5, 18, 24). Hence, despite being annotated as an exported PEXEL protein, this phenotype of the *pv6* mutant implies that PV6 performs an essential function prior to the time at which protein export is required. We followed the development of the mutant parasites, starting from the invasion of the merozoite, to gain a more detailed understanding of the timing of the appearance of this phenotype and determine whether the parasites undergo a temporary delay in development or suffer a terminal block in development. We synchronized the PfBLD529 parasites in which the *pv6* gene could be deleted by the addition of rapamycin (36, 40), induced deletion of the *pv6* gene approximately 30 h after invasion, and subsequently synchronized parasite egress using the cGMP-dependent protein kinase inhibitor compound 2 (42). A visual inspection of Giemsa-stained schizonts treated with rapamycin or DMSO did not reveal any obvious difference in morphology (Fig. 1A), indicating that the loss of the *pv6* gene does not affect the development of late-stage parasites and merozoites. After the removal of compound 2, the diameter of the re-invaded rings was measured. The rapamycin-treated rings were significantly smaller at 1, 8, and 16 h post-invasion. Interestingly, the size of the mutant rings did not seem to change during the remainder of the period during which the parasite was followed (Fig. 1B), consistent with the prior finding that parasites lacking PV6 stop developing at an early stage after erythrocyte invasion and do not appear to assume the classic ring morphology, even at later stages in the intraerythrocytic cycle (36). As previous experiments have shown that parasites lacking PV6 fail to develop over several cycles, this lack of development is likely a terminal phenotype (36). Therefore, we conclude that PV6 functions prior to the requirement for protein export.

As PV6 functions very soon after invasion, it is very likely present in merozoites at the time of invasion. We, therefore, carried out colocalization studies using markers of apical organelles to determine its localization. This revealed that the protein is indeed present in merozoites, but we were not able to detect consistent colocalization of PV6 with the standard markers of apical organelles; no colocalization was detected with markers of the microneme (AMA1), rhoptry (RAP1), rhoptry neck (RON4), or dense granules (RESA) (Fig. 1C; Fig. S1B). As RESA and PV6 appeared to be closely positioned together, we determined whether PV6 colocalizes with EXP2, another dense granule marker. This also did not colocalize with PV6, even when localization was determined in merozoites (Fig. S1C).

### PV6 is cleaved by Plasmepsin V but not exported

PV6 was initially identified as a PEXEL protein, and subsequent studies have identified it as a substrate of PM V using mass spectrometry (45, 46). However, as the phenotype of the *pv6* mutant indicates that the protein does not function as an exported protein, unlike other known PM V substrates, we aimed to verify biochemically that PV6 is indeed cleaved by PM V. For this, we treated parasites with the PM V inhibitor WEHI-1252601 (601) for 3 h, starting approximately 30 h after invasion; at this time point, PV6 is already produced (37), and protein export has commenced, decreasing the chance that the addition of the inhibitor has a detrimental effect on the parasites. However, some PV6 that has already been processed will be present in these samples (Fig. 2A, upper panel, time 0). Immunoblotting revealed the appearance of a band with the expected molecular mass of full-length, uncleaved PV6 (53.6 kD) in the extracts of parasites treated with the inhibitor (Fig. 2A, upper panel); no size shift was detected for EXP2, a protein that is cleaved by signal peptidase, in these samples (Fig. 2A, lower panel). These results confirm that PV6 is indeed a PM V target.

We next determined the localization of PV6 after invasion using immunofluorescence. This revealed that PV6 remains in the PV (Fig. 2B) even in cells where the exported protein RESA has been exported and has bound to the host cell cytoskeleton, with no detectable RESA remaining in the PV (Fig. 2B; Fig. S2A); no PV6 was detected in the erythrocyte. The protein was detected as the characteristic arc of staining (Fig. 2B, top and bottom panels), although some of the protein may also be present within the parasite (Fig. 2B, bottom panel; Fig. S2B, bottom panel). The localization of PV6 was similar to that of EXP2, although interestingly, the signals do not seem to overlap completely (Fig. S2B), indicating that PV6 may be, at least in part, localized outside of the PV/PVM domains containing EXP2 previously identified (47).

However, it is often difficult to visualize soluble exported proteins in the cytoplasm of the host cell, especially at the early stages of the intraerythrocytic cycle when soluble proteins are greatly diluted in the host cell cytoplasm compared to the PV. We, therefore, determined biochemically whether the protein is retained in the PV using selective permeabilization of the host cell and PVM with streptolysin O and saponin, respectively (48–50). Treatment of erythrocytes infected with trophozoite-stage parasites with PBS, which leaves the erythrocyte intact, or SLO, which perforates the erythrocyte membrane and thereby releases exported proteins into the supernatant while leaving the PVM intact, did not release PV6, even though the soluble exported protein HRPII was released (Fig. 2C). In contrast, treatment of the parasite with saponin, which lyses the erythrocyte membrane and the PVM, released PV6, along with the previously characterized PV protein PV1 (51, 52). This further indicates that PV6 is a PV-resident protein. Collectively, these results show that PV6 is a PM V target that remains in the PV; therefore, we propose the name PV6 for this protein to reflect its subcellular localization.

### An AMA1-signal sequence-HA_3_-PV6 fusion can replace the function of native PV6

Previous studies revealed that, when overexpressed using a Cam promoter, a PV6-GFP fusion protein can be detected in the host cell cytosol (38). Although the results above show that PV6 is expressed and appears to function in the PV, we could not completely rule out potential additional functions of PV6 in the erythrocyte. We, therefore, produced a version of the gene that encodes a protein that is cleaved by signal peptidase, rather than PM V, to replace the native gene. For this, we fused the conserved region of PV6 starting from codon 122, including the START domain and the regulatory region in the C-terminus but lacking the PEXEL to sequence encoding the AMA1 signal sequence (Fig. 3A). In addition, we included sequence encoding a 3xHA tag (HA_3_) between the AMA1 signal sequence and the PV6 sequence, producing an AMA1-SS-HA_3_-PV6 fusion (Fig. 3A). As a control, we generated a fusion gene that contains the first 77 codons of PV6, including the PEXEL and 12 downstream codons, fused to sequence encoding the HA_3_ tag and PV6 starting from codon 122, producing a PV6-HA_3_-PV6 fusion. These fusion genes were used to replace the native *pv6* gene using Cas9-mediated gene replacement, leading to rapid and efficient integration; no wild-type version of the gene could be detected by PCR in the transfected parasite populations (Fig. 3B). The genetically modified parasites expressed the fusion proteins, as indicated by anti-HA immunoblots (Fig. 3C). Of note, the modification of the PV6 N-terminal region leads to the production of slightly smaller proteins, as displayed in the anti-PV6 blot (Fig. 3C).

To ascertain that the AMA1-SS-HA_3_-PV6 fusion is not cleaved by PM V, we treated wild-type parasites and parasites expressing the AMA1-SS-HA_3_-PV6 fusion protein with the PM V inhibitor WEHI-1252601. Whereas a band of higher molecular mass was detected in extracts of wild-type PV6 parasites that had been treated with the inhibitor, no such band was detected in extracts of parasites expressing AMA1-SS-HA_3_-PV6 (Fig. 3D). Hence, AMA1-SS-HA_3_-PV6 is not a PM V substrate. We furthermore determined the localization of the fusion proteins by fluorescence microscopy and biochemically, which revealed that both fusions are present almost exclusively in the PV (Fig. 3E; Fig. S3).

As the genetically modified parasites were obtained quickly after the transfections (within 3–4 weeks) and without competing wild-type parasites, the growth rate of the parasites producing the fusion protein did not appear to be affected. Growth assays confirmed that the AMA1-SS-HA_3_-PV6 and PV6-HA_3_-PV6 parasites do indeed grow at the same rate as the parent 3D7 parasite line (Fig. 3F). Hence, the fusion protein that is targeted to the ER by a signal sequence, but is not cleaved by PMV, supports parasite growth. This indicates that cleavage by PM V is not essential for the correct trafficking and function of the protein.

### Residue 4 of the PEXEL determines export of PV6

**Fig 4:**
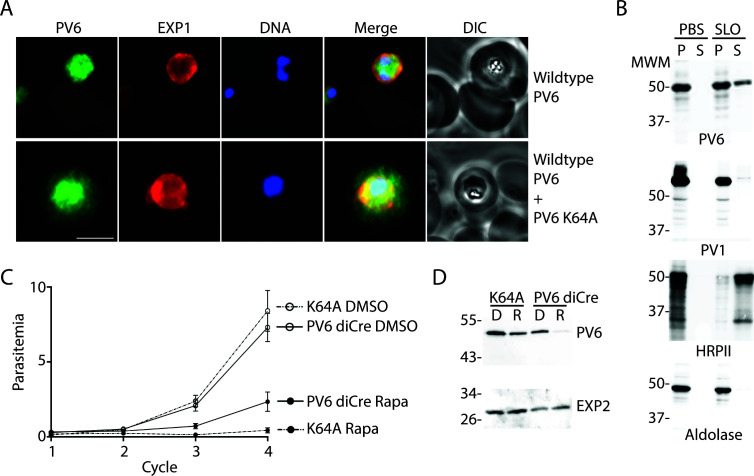
Localization and function of a PV6 K64A mutant. (**A**) Immunofluorescence assays of parasites expressing wild-type PV6 or wild-type PV6 plus PV6 K64A. The PVM was visualized with an anti-EXP1 antibody. PV6 was detected in the erythrocyte cytosol only in erythrocytes infected with parasites expressing the PV6 K64A mutant. See Fig. S3 for additional images. The scale bar represents 5 µm. (**B**) Selective permeabilization of erythrocytes infected with wild-type or parasites expressing the K64A mutant. Infected erythrocytes were treated with PBS or Streptolysin O (SLO) and pelleted. The pellet and supernatant were subjected to SDS-PAGE and immunoblotted with the indicated antibodies. Note the release of PV6 in the supernatant of the permeabilized erythrocytes infected with parasites expressing PV6 K64A, whereas nearly all of the PV marker PV1 is retained in the parasite. (**C**) Growth assay of PV6 diCre and parasites expressing PV6 K64A after treatment with DMSO or rapamycin to remove the native *pv6* gene. Growth assays were set up in triplicate; error bars indicate the error of mean but are, in several cases, too small to protrude from the symbol. (**D**) Anti-PV6 (upper panel) and anti-EXP2 (lower panel) immunoblots of parental (PV6 diCre) and transfected parasite (K64A mutant line). Of note, PV6 expression is lost after rapamycin treatment in the parental line. When the same treatment is carried out on the mutant line, the protein signal is only reduced, reflecting the continued expression of the K64A mutant protein from the *pfs47* locus by the transgenic parasite.

The PEXEL of PV6, RILKE, is unusual in that the fourth residue is lysine. Although the PEXEL is usually denoted as RxLxE/Q/D [with a K allowed at the first position in some cases (13)], previous studies have shown that the fourth residue affects the export of PEXEL proteins (31, 35). Hence, we postulated that this residue may prevent export into the host cell. We therefore replaced the codon encoding the K residue with a codon encoding an A (K64A mutation), which is present at the fourth position of the PEXEL of many exported proteins, including the well-characterized exported protein KAHRP (53), and introduced this mutated gene, controlled by the native *pv6* promoter, at the *pfs47* locus (41) in the PfBLD529 (PV6-diCre) background, producing a merodiploid (Fig. S4A, Fig. S4B). By immunofluorescence microscopy, using rabbit anti-PV6 antibodies, PV6 was detected in the cytosol of erythrocytes infected with parasites that produce PV6 with the K64A mutation (Fig. 4A, upper panel), in contrast to wild-type parasites, where the protein was detected only within the parasite and the PV, as identified by staining with the PVM marker EXP1 (Fig. 4A, lower panel). We repeated the experiment with a monoclonal mouse anti-PV6 antibody, using PV1 as a marker for the PV, and once again, we detected PV6 only in the parasite and the PV in untransfected parasites but detected PV6 also in the host cell cytosol in erythrocytes infected with parasites expressing the PV6 K64A mutant (Fig. S4C). We also determined the localization of PV6 in erythrocytes infected with PfBLD529 (PV6-diCre) parasites that express the PV6 K64A mutant that had been treated with rapamycin to remove the native *pv6* gene, leaving the K64A mutant as the only form of PV6 in the parasite. In these parasites, anti-PV6 staining was found within the erythrocyte and the parasite itself, indicating that it is exported (Fig. S4D). These results indicate that the change in the N-terminal residue from K to A allows PV6 to be exported.

To verify the microscopy results biochemically, we again performed differential lysis of infected erythrocytes using saponin and SLO. PV6 was readily detected in the supernatant of SLO-treated erythrocytes that were infected with parasites expressing the version of PV6 with the K64A mutation, indicating that the mutated version of the protein is exported (Fig. 4B). As these parasites also express the native version of the protein, PV6 was also detected in the parasitophorous vacuole (Fig. 4B, SLO pellet). As expected, the exported protein HRPII was also readily detected in SLO supernatant of both strains, whereas the PV protein PV1 protein was detected almost entirely in the pellet of the SLO-treated parasites of both strains (Fig. 4B).

To test whether PV6 carrying the K64A mutation can support growth, we compared the growth rate of the K64A parasites with that of the parent parasite line, PfBLD529 (PV6 diCre), in which the native *pv6* gene can be removed by treating the parasites with rapamycin. After DMSO treatment, the PfBLD529 (PV6-diCre) parasites that express the PV6 K64A grew at the same rate as parasites of the PfBLD529 (PV6-diCre) parental strain, suggesting that the expression of K64A in the presence of WT PV6 does not induce a detectable dominant negative impact resulting in a growth defect. However, treatment with rapamycin leads to a severe growth defect, indicating that PV6 K64A does not support parasite growth (Fig. 4C).

As expected, immunoblot analysis confirmed the almost complete loss of PV6 expression in the parental line treated with rapamycin, whereas only a reduction is observed in the treated mutant (Fig. 4D). Therefore, the signal detected by the anti-PV6 antibodies corresponds most likely to the K64 PV6 protein encoded at the *pfs47* locus, as suggested by the diagnostic PCR analysis revealing a complete excision of the native *pv6* after rapamycin treatment of the K64A mutant (Fig. S4B).

Together, these results show that the native PV6 is not targeted for export but instead retained in the PV, owing to the K residue at its N-terminus.

### Analysis of the putative N-termini of PEXEL proteins, PNEPs, PV proteins, PVM proteins, and parasite surface proteins

The finding that the K residue at the N-terminus of PV6 does not allow export of the protein prompted us to examine in more detail how often each amino acid is present at the fourth position of the PEXEL in proteins predicted to be exported and whether a K residue is present at the fourth position of known exported proteins. As has been noted previously, amino acids with small, hydrophilic side chains are common in this fourth position (11, 12, 31, 35, 53). Consistent with this, when we determined the frequency at which each amino acid is observed at the fourth position in predicted exported proteins in three different published exportomes (the Boddey, Sargeant, and van Ooij exportomes), we found that most often, a small, polar amino acid is found at this position (11, 12, 15, 53). Overall, we found that 10 different amino acids are observed at the fourth position of verified exported proteins (Table 1). Five residues, A, C, S, T, and Y, are very common at this position (Fig. S5), in part because they frequently occur in the large protein families STEVOR and RIFIN, although these residues are also present in many proteins for which export has been verified that are not part of large families, including KAHRP and PfEMP3 (A) (53, 54) and several FIKK kinases, MESA, REX3, and REX4 (S) (13, 55–57). Export of proteins with other less common residues at position 4, including H (HRPII and HRPIII) (58), G (RIFIN PFI0050c/PF3D7_0901000, PTP2 and the *Plasmodium vivax* protein PHIST CVC-8195/VX_093680) (59–62), L (FIKK7.1) (57), N (GARP, FIKK9.7, and FIKK13) (57, 63), and V (GEXP18) (64) has also been observed. No known exported protein contains a large charged residue at the fourth position (such as K), although each exportome predicts several such proteins. However, many of these predicted proteins have been shown or are predicted to be targeted to intracellular locations within the parasite or lack an annotated signal peptide or transmembrane domain and, therefore, are unlikely to be exported proteins (Table S3). Five amino acids were not observed in the fourth position of the PEXEL proteins: D, M, P, Q, and W. Based on this, we categorized the amino acids into “common, confirmed” (A, C, S, T, and Y), “uncommon, confirmed” (G, H, L, N, and V), “not confirmed, unlikely” (E, F, K, and R), “not confirmed” (I), and “not observed” (D, M, P, Q, and W) (Table 1, also see Table S4).

**TABLE 1:** Occurrence of all amino acids in position 4 of the PEXEL in proteins predicted to be exported in the indicated exportomes^*a*^

Category	Amino acid at fourth position	Boddey [number of proteins (%)]		Sargeant [number of proteins (%)]		van Ooij [number of proteins (%)]		Confirmed export
Common, confirmed	A	83	22.3	82	20.7	70	25.3	FIKK kinase 10.1, GBP130, EMP3, STEVOR, KAHRP
	C	92	24.7	93	23.5	87	31.4	Rifin (PFA0745w/PF3D7_0115300)
	S	105	28.2	114	28.9	71	25.6	FIKKs, MESA, REX3, REX4, RIFIN
	T	24	6.4	26	6.6	18	6.5	FIKK4.1, Hyp1 (PF3D7_0113300)
	Y	21	5.7	26	6.6	9	3.3	FIKK11, RESA
	**Total**	**325**	**87.4**	**341**	**86.1**	**255**	**92.1**	
Uncommon, confirmed	G	7	1.9	5	1.3	5	1.8	Rifin, PTP2, Pf3D7_0801000 (PfPHIST_0801), PVX_093680 (PHIST CVC8195)
	H	3	0.8	2	0.5	2	0.7	HRPII, HRPIII
	L	11	3.0	10	2.5	1	0.4	FIKK7.1
	N	5	1.3	9	2.3	0	0	GARP, FIKK
	V	7	1.9	8	2.0	4	1.44	GEXP18
	**Total**	**33**	**8.9**	**34**	**8.6**	**12**	**4.3**	
None confirmed, unlikely	E	1	0.3	3	0.8	0	0	
	F	4	1.1	4	1.0	3	1.1	
	K	5	1.4	5	1.3	4	1.4	
	R	0		0		1	0.4	
	**Total**	**10**	**2.7**	**12**	**3.0**	**8**	**2.9**	
Not confirmed	I	**4**	**1.1**	**9**	**2.3**	**2**	**0.7**	
Not observed	D	0	0	0	0	0	0	
	M	0	0	0	0	0	0	
	P	0	0	0	0	0	0	
	Q	0	0	0	0	0	0	
	W	0	0	0	0	0	0	
Total		372	100	396	100	277	100	

^*a*^ For references for confirmation of protein export, please see text. For further information, please see Fig. S5.

As PEXEL-negative exported proteins are also exported through the PTEX (3), we furthermore examined the N-termini of several PNEPs and the members of the SURFIN family (Table 2). As the N-terminal methionine residue is removed by methionine aminopeptidase when it is followed by a small residue (primarily G, A, S, T, C, P, and V) (65), we focused on the second residue of the protein. We found that most PNEPs contain a small amino acid after the start methionine and, hence, are likely to be processed by methionine aminopeptidase. PF3D7_1148900/PF11_0505 is a PNEP that contains a residue following the M residue that has not been detected at position 4 of the PEXEL of exported proteins and that is not thought to be removed (N and E), making it likely to retain its start M. Although export was detected for PF11_0505, the proportion of the protein that was exported was much lower than that of the other PNEPs (26). However, this may have been related to the presence of the GFP tag, as a GFP fusion of PF11_0505 with a different linker is exported more robustly. Furthermore, REX2, which is predicted to have a K orY residue at the position following the N-terminus, has been shown to be processed more extensively; the N-terminal peptides that have been experimentally verified are LAEIFSSGK and AEIFSSGK (28). Hence, it is possible that further processing in the ER can generate different N-termini, which can affect its export.

**TABLE 2:** N-termini of known PNEPs and the SURFIN family^*b*^

Gene ID	Protein name (former ID)	N-terminus	Methionine amino peptidase	New N-terminus	
PF3D7_0501300	SBP1	M**C**SAARAFDFFTDLA	✓	C	
PF3D7_1370300	MAHRP1	M**A**EQAAVQPESVPTV	✓	A	
PF3D7_1353200	MAHRP2	M**Q**PCPYDVYNQINHV	✗	Q	
PF3D7_0935900	REX1	M**A**DYSSNEEETPKEE	✓	A	
PF3D7_0936000	REX2^*a*^	M**K**M**Y**LAEIFSSGKES	✗	K?	Y?
PF3D7_1149000	Pf332	M**C**SAARAFDFFTDLA	✓	C	
PF3D7_0702400	SEMP1/PF07_0007	M**S**QPQKQQNEEGAAT	✓	S	
PF3D7_0702500	PF07_0008	M**A**YPLLEDDLRSIRV	✓	A	
PF3D7_1334700	MSP7-like/PF13_0194	Signal sequence predicted 27 and 28: SQS-VN	NA	V	
PF3D7_1404800	PF14_0045	Signal sequence predicted 21 and 22: TWA-LF	NA	L	
PF3D7_0830500	TryThrA/PF08_0003	M**N**LEQFKNINKDLAT	✗	N	
PF3D7_1148900	PF11_0505	M**E**AEKKEEKQEKSVK	✗	E	
PF3D7_0113100	SURFIN 1.1	M**E**QIGIANNIFTEIG	✗	E	
PF3D7_0113600	SURFIN 1.2	M**H**FVVEFQDWIKEPK	✗	H	
PF3D7_0115000	SURFIN 1.3	M**A**VKISTSVYKKRIS	✓	A	
PF3D7_0402200	SURFIN 4.1	M**H**FVVELDNTGDDF	✗	H	
PF3D7_0424400	SURFIN 4.2	M**L**FVVELDSRLEKSAD	✗	L	
PF3D7_0831100	SURFIN 8.1	M**A**IISSIPNYRKGLNP	✓	A	
PF3D7_0830800	SURFIN 8.2	M**L**MVIEFDDPIKEPS	✗	L	
PF3D7_0800700	SURFIN 8.3	M**A**VEMNTPVSKKGVS	✓	A	
PF3D7_1301800	SURFIN 13.1	M**A**SYIKLNIPLKPKK	✓	A	
PF3D7_1477600	SURFIN 14.1	M**K**VKKDNPNYKKRIN	✗	K	

^*a*^ Experimental verification of the N-terminus of REX2 revealed the presence of peptides starting with an A or L.

^*b*^ Indicated are the N-termini of the protein, whether the start methionine is likely to be removed and the expected N-terminal residue after processing by methionine aminopeptidase. NA, not applicable.

Considering this bias toward certain N-terminal residues in exported proteins, we next determined whether these residues are also common at the N-termini of proteins that are not exported. Interestingly, an analysis of the predicted N-termini of 73 PV, PVM, and parasite surface proteins (which are processed by signal peptidase rather than PM V) revealed that frequently, the predicted N-terminal residue is a large, charged residue, whereas small, polar residues are less common (Table 3; Table S5)—over 43% (30/70) of these proteins have a predicted N-terminal residue that is in the “none confirmed, unlikely” category of PEXEL proteins (i.e., an E, F, K, or R residue) and the second largest category (22/70) is “uncommon, confirmed” (i.e., a G, H, L, N, or V residue). Only 20% (14/70) of the proteins contained a predicted N-terminal residue that is common in the fourth position of exported PEXEL proteins [“common, confirmed” (i.e., an A, C, S, T, or Y residue)]. Included among these are the exported proteins Clag3.1 (S) and MSRP6 (S) (5, 66, 67) [although there are different reports about the requirement of the PTEX for the export of Clag3 and the RhopH proteins (5, 68)]. Note that in three proteins, no signal sequence was predicted by SignalP. Interestingly, although the residue in the fourth position of the PEXEL is not conserved among PV6 orthologs in other *Plasmodium* species, nearly all contain a large, charged residue at this position (Table S2); only in the *Plasmodium gallinaceum* ortholog were we unable to find a canonical PEXEL sequence.

**TABLE 3:** Frequency of predicted N-terminal amino acids of secreted apical organelle proteins, parasite surface proteins, parasitophorous vacuole proteins, and parasitophorous vacuole membrane proteins^*a*^

Category	Frequency (%)
Common, confirmed	20.0
Uncommon, confirmed	31.4
None confirmed, unlikely	43.4
Not confirmed	4.3
Not observed	1.4

^*a*^ For a full list of proteins, please see Table S5.

These results are consistent with previous findings that the N-terminal residue of proteins reaching the PV and exposed to the PTEX likely contributes to whether the protein will be exported. It also shows that the three pathways that the parasite uses to translocate and process proteins into the ER for transport to the PV—signal peptidase-mediated processing, PM V-mediated processing, and post-translational transfer into the ER (in the case of PNEPs)—tend to expose different residues at new N-termini, potentially explaining how the different pathways promote export or retention in the PV.

### At least one additional Plasmepsin V target is essential for parasite development immediately after invasion

**Fig 5:**
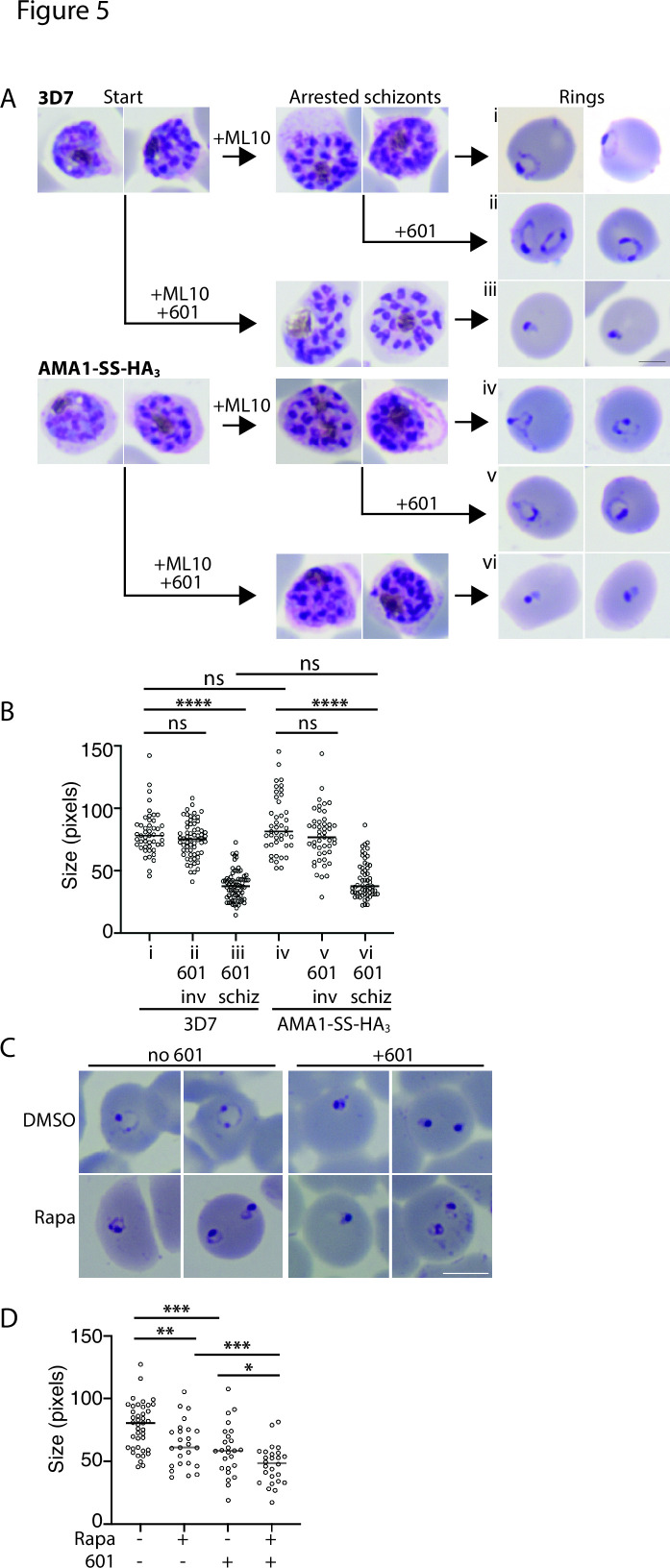
Developmental arrest induced by Plasmepsin V inhibitors after invasion. (**A**) Synchronized cultures of 3D7 and AMA1-SS-HA_3_-PV6 parasites were treated with ML10 when the parasites were in the early schizont stage, and half of the culture was simultaneously treated with the PM V inhibitor WEHI-1252601 (601) (Start). ML10 and 601 were removed when most schizonts appeared arrested (Arrested schizonts). The culture treated only with ML10 was split into two, and one half was treated with 601. Ring formation was determined 1 h after the removal of ML10 (Rings). The scale bar on the right-hand side of panel iii represents 2.5 µm. (**B**) Quantitation of parasite diameter size in samples i–vi in panel A. Indicated is the time the PM V inhibitor 601 was added; inv, after removal of ML10 from arrested schizonts; schiz, at start in Panel A (and removed with ML10). Data are based on the measurement of at least 40 rings. The Mann–Whitney *U* test was performed for statistical analysis; ns: not significant, ****P < 0.0001. (**C**) Treatment of parasites containing a floxed *pv6* locus with rapamycin and 601, as indicated. Note the more compact shape of the parasites treated with 601 compared to the rapamycin-treated parasites. The scale bar represents 5 µm. (**D**) Quantitation of parasite sizes from the experiment shown in panel C. Data are based on the measurement of at least 20 rings. The Mann–Whitney *U* test was performed for statistical analysis: ns: not significant, *P < 0.05, **P < 0.01, ***P < 0.001, and ****P < 0.0001.

A previous study revealed that treatment of malaria parasites with a PM V inhibitor late in the erythrocytic cycle leads to a phenotype very early in the next erythrocytic cycle, much earlier than the ring–trophozoite stage arrest detected in mutants deficient in protein export (3, 5, 23, 24). As AMA1-SS-HA_3_-PV6 relies on signal peptidase rather than PM V for its processing, it afforded the opportunity to determine whether the ring-stage phenotype detected after treatment with PM V inhibitors is the result of the lack of processing of PV6 or whether additional PEXEL proteins are required for early ring-stage development. We treated synchronized wild-type 3D7 parasites and parasites producing AMA1-SS-HA_3_-PV6 with the PM V inhibitor 601 during the schizont stage (Fig. 5A) and inhibited egress with ML10 to control the timing of invasion (39, 44). The development of the schizonts did not appear to be affected in either parasite strain by the presence of 601, as judged by observation of Giemsa-stained parasites (Fig. 5A). We then removed the ML10 and 601 to allow egress and invasion. In the case of the parasites treated only with ML10, we added 601 to half of the culture to determine whether 601 affects invading parasites and early rings. Parasites treated only with ML10 and parasites exposed to 601 at the time of removal of ML10 [approximately 20 minutes before egress and subsequent invasion (39)] developed into normal ring-stage parasites. In contrast, parasites that had been treated with 601 during schizogony (but invaded in the absence of the inhibitor) showed a very severe developmental defect. Rather than forming the characteristic ring shapes, parasites of both strains were detected as very compact spots (Fig. 5A). Measurement of the size of the parasites showed that there was a significant size difference between rings formed by parasites treated with 601 as schizonts (Fig. 5A, panel iii, and Fig. 5B) and untreated parasites and parasites treated during egress (Fig. 5A, panels i and ii, respectively, and Fig. 5B). No difference in the effect of the inhibitor was detected between wild-type parasites and parasites that encode the AMA1-SS-HA_3_-PV6 protein (Fig. 5B), indicating that even though the AMA1-SS-HA_3_-PV6 parasites produce a version of PV6 that does not rely on PM V for its processing (Fig. 3D), the parasites were sensitive to the inhibition of PM V. Interestingly, the phenotype of wild-type parasites arrested by 601 did not phenocopy the *pv6* mutant (Fig. 1A); the inhibitor-treated parasites formed small, compact parasites (Fig. 5A, panels iii and vi), as also previously reported (23), whereas parasites lacking PV6 formed slightly more expanded rings, often with a translucent center (Fig. 1A). Furthermore, the phenotype displayed by the treated parasites did not phenocopy that of parasites in which the related protease Plasmepsin IX or Plasmepsin X was inactivated; the lack of activity of those proteases causes an egress defect (Plasmepsin X) or invasion defect (Plasmepsin IX) and thereby prevents invasion (69–71). Hence, although we cannot completely rule out that the inhibitor has additional targets, the phenotype observed is not consistent with the inhibition of Plasmepsin IX and Plasmepsin X.

To compare the phenotypes of parasites lacking PV6 and parasites in which PM V is inhibited directly, we repeated the experiment, treating PV6-diCre parasites carrying the floxed version of *pv6* with DMSO and rapamycin and with 601. This showed once again that parasites lacking PV6 form small, round rings that often have a small translucent center. In contrast, parasites treated with 601 showed a more severe phenotype, with more compact parasites that, in many cases, did not have a clear center (Fig. 5C). Quantitation of the size of the parasites further underscored the difference in size between the wild type, the mutant, and the inhibitor-treated parasites (Fig. 5D).

Together, the findings that parasites expressing AMA1-SS-HA_3_-PV6 remain sensitive to a PM V inhibitor, with a similar phenotype to that of wild-type parasites after invasion, and the difference in the morphology of parasites treated with the PM V inhibitor and parasites lacking PV6 suggest the possibility that at least one additional non-exported PM V substrate functions very soon after invasion.

## DISCUSSION

In this study, we show that despite being cleaved by PM V, the *P. falciparum* phospholipid transfer protein PV6 is retained and functions in the PV. This is the first demonstration that a native PM V substrate in malaria parasites is not exported into the host cell and shows that cleavage by PM V does not always, *per se,* target a protein for export. Furthermore, we show that the fourth residue of the PEXEL in PV6, a K, which becomes the N-terminal residue after cleavage by PM V, determines the localization of the protein; when this residue is changed to an A—a residue found in the fourth position of several exported proteins, including KAHRP (53)—the protein is exported. Previous studies have shown that altering this position in exported proteins can severely decrease the fraction of the protein that is exported without affecting cleavage by PM V (31, 35). However, PV6 is the first native PM V substrate identified that has a demonstrated essential function in the PV. A recent study by Fierro et al. revealed that the *P. falciparum* protein UIS2 is also cleaved by PM V but is not exported (72); notably, the N-terminal residue of this protein after processing is a D, whereas the N-terminal residue of the *Plasmodium berghei* orthology is a Q (export of this ortholog was not determined in the blood stages, however) (45, 72). These findings indicate that processing of a protein by PM V does not inevitably result in protein export and that processing by PM V and recognition of proteins for export seem to be two separate events that are unlikely to be mechanistically coupled.

Our results furthermore provide additional support for the finding that the main determinant of protein export is the N-terminal residue (31, 35), as long as this is followed by a flexible region of approximately 10 amino acids and the protein can be unfolded by HSP101 (29, 30, 32), although additional residues following the N-terminus also influence protein export (26, 35, 73). PEXEL proteins for which export has been experimentally verified most commonly contain a small, polar residue at the fourth position of the PEXEL (Table 1) (11, 12). Furthermore, the PNEPs analyzed in this study contain, in many cases, a small, polar residue in the second position—these residues are also favored by methionine aminopeptidase for the removal of the N-terminal M residue and will, therefore, likely become the N-terminus after processing by the peptidase. In contrast, processing of parasite surface proteins, PV proteins, PVM proteins, and apical organelle proteins by signal peptidase is predicted to expose, in many cases, a large, charged residue at the newly formed N-terminus—residues that are not found in known exported proteins. One notable exception is the exported protein MSRP6 (5, 26, 67), in which an S is predicted to become the N-terminal residue after processing by signal peptidase. Based on this, we propose a model in which the N-terminal region of a protein with a small, polar residue at the extreme N-terminus is recognized by the PTEX (potentially HSP101 or an accessory factor) and thereby targets these for export, regardless of the process by which the N-terminus has been generated. Proteins that feature a large, charged residue at the N-terminus, such as many surface proteins, PV proteins, and PVM proteins that are processed by signal peptidase and, as shown here and by Fierro et al. (72), a subset of PM V substrates are not recognized and hence are not exported, thereby preventing improper removal of proteins from either the surface of the parasite or the PV or clogging the PTEX with parasite surface proteins. The fate of proteins that feature at their N-terminus an amino acid that is present in exported and surface proteins may be influenced by the residues that follow the N-terminal residue, as these also play an important role in determining the localization of parasite proteins (31, 35, 73).

In previous studies, PV6 has been identified as an exported protein, and data indicated that a GFP fusion was indeed exported, although bright fluorescence was also present in the parasite itself (37, 38). However, in these cases, the expression of the protein was controlled by the *cam* promoter, which is stronger than the native *pv6* promoter. When the native *pv6* promoter was used in one of these studies, the protein was detected in a pattern that was consistent with a PV localization. Hence, when expressed at native levels, PV6 does not appear to be exported (37).

Together, our results uncouple processing by PM V from protein export and indicate that the role of PM V in *Plasmodium* parasites is broader and likely more ancient than processing exported proteins, as has been postulated previously (74). Its role, therefore, may be more similar to that of its *Toxoplasma gondii* ortholog, the aspartic protease ASP5, than previously thought. One notable difference between the two organisms is that nearly all ASP5 substrates function in the PV and the PVM (75, 76), whereas all PM V substrates were thought to be exported. Our findings that a PV protein is cleaved by PM V and that there is at least one other PM V target that does not function in the cytosol of the host erythrocyte may indicate that in *Plasmodium* parasites, PM V also functions to process PV and PVM proteins. As orthologs of PM V are conserved among many Apicomplexan parasites, we postulate that PM V evolved in Apicomplexan parasites to cleave PV or PVM proteins and that the role of PM V in processing exported proteins evolved subsequently in *Plasmodium* parasites. It is of note that conserved PEXEL proteins (including orthologs of PV6) generally contain a large, charged residue at position 4 of the PEXEL (38). It is possible that, contrary to what has been reported previously (38), most, if not all, conserved PEXEL proteins function as non-exported proteins, indicating that their functions may have arisen prior to the development of the export machinery in malaria parasites. Why the parasite has evolved to use two proteases, PM V and signal peptidase, to process proteins that enter the secretory pathway remains to be discovered.

## SUPPLEMENTAL MATERIAL

Figure S1 mbio.01223-23-s0001.pdf Additional images of IFA in Fig. 1B.

Figure S2 mbio.01223-23-s0002.pdf Immunofluorescence assay PV6 with RESA and EXP2.

Figure S3 mbio.01223-23-s0003.pdf Immunoblot SLO lysis of AMA1-HA-PV6 fusion and PV6-HA-PV6.

Figure S4 mbio.01223-23-s0004.pdf Export of PV6 K64A mutant.

Figure S5 mbio.01223-23-s0005.pdf Overview of exportomes.

Supplemental reference mbio.01223-23-s0006.docx Reference for supplemental material.

Supplemental tables mbio.01223-23-s0007.xlsx Tables S1–S5.

ASM does not own the copyrights to Supplemental Material that may be linked to, or accessed through, an article. The authors have granted ASM a non-exclusive, world-wide license to publish the Supplemental Material files. Please contact the corresponding author directly for reuse.
