# Supplementary material for: Identification of a non-exported Plasmepsin V substrate that functions in the parasitophorous vacuole of malaria parasites
Source: mBio. 2023 Dec 11;15(1):e01223-23. doi: 10.1128/mbio.01223-23 (PMC10790765; doi:10.1128/mbio.01223-23)
Supplement: Figure S1 — Additional images of IFA in Fig. 1B. [file mbio.01223-23-s0001.pdf]

Supplementary Figure 1

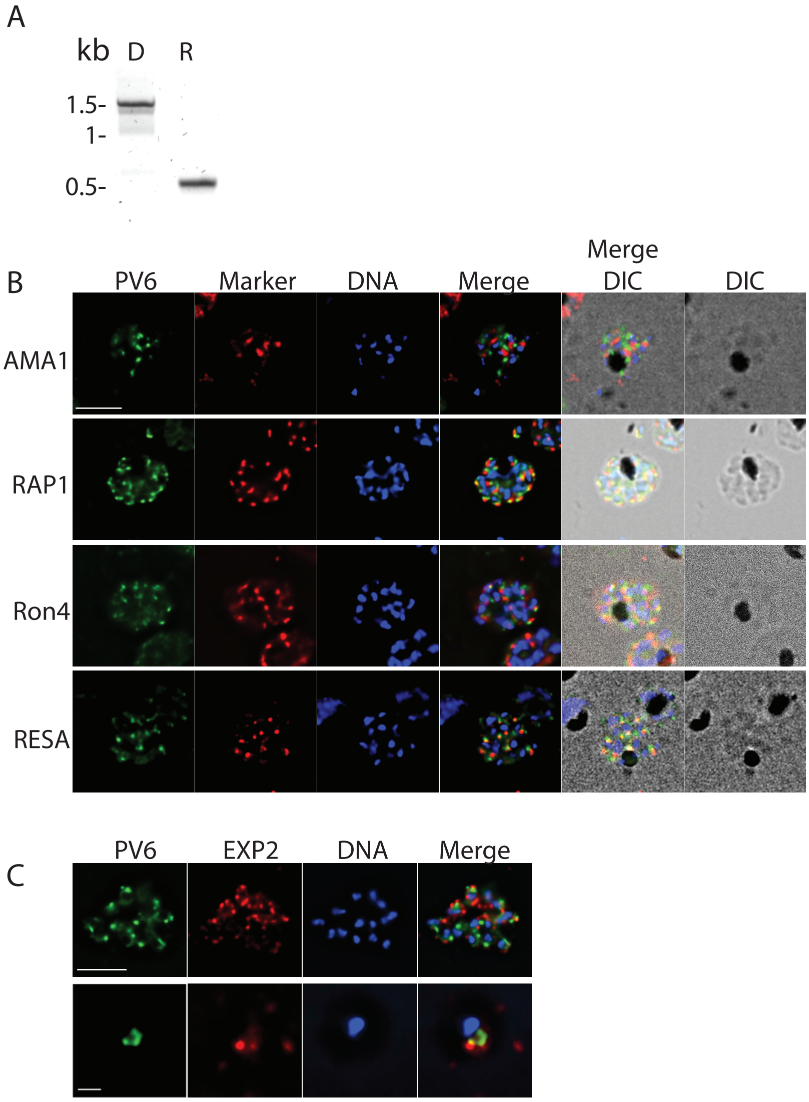

Supplementary Figure 1. Excision PCR of parasites with floxed *pv6* (*pfa0210c/PF3D7_0104200*) locus and costaining of parasites with antibodies against dense granule markers markers.
A). Excision PCR of the parasite line containing the floxed *pv6* locus used in Figure 1, showing the pv6 locus after parasites have been treated with DMSO (D) or rapamycin (R). See Hill *et al.* for outline of PCR strategy (1).
B) Colocalization of PV6 (green) with apical organelle markers (red) and DNA as detected with Hoechst staining. These panels show the individual channels of the images shown in Figure 1C.
C) Immunofluorescence assay of a lysed late-stage schizont (top) and free merozoite (bottom) with antibodies against PV6 (green) and the dense granule marker EXP2 (red). The DNA was stained with Hoechst 33342 and is shown in blue. Scale bar top images: 5 µm; scale bar bottom images: 1 µm.
