# Supplementary material for: Identification of a non-exported Plasmepsin V substrate that functions in the parasitophorous vacuole of malaria parasites
Source: mBio. 2023 Dec 11;15(1):e01223-23. doi: 10.1128/mbio.01223-23 (PMC10790765; doi:10.1128/mbio.01223-23)
Supplement: Figure S2 — Immunofluorescence assay PV6 with RESA and EXP2. [file mbio.01223-23-s0002.pdf]

Supplementary Figure 2

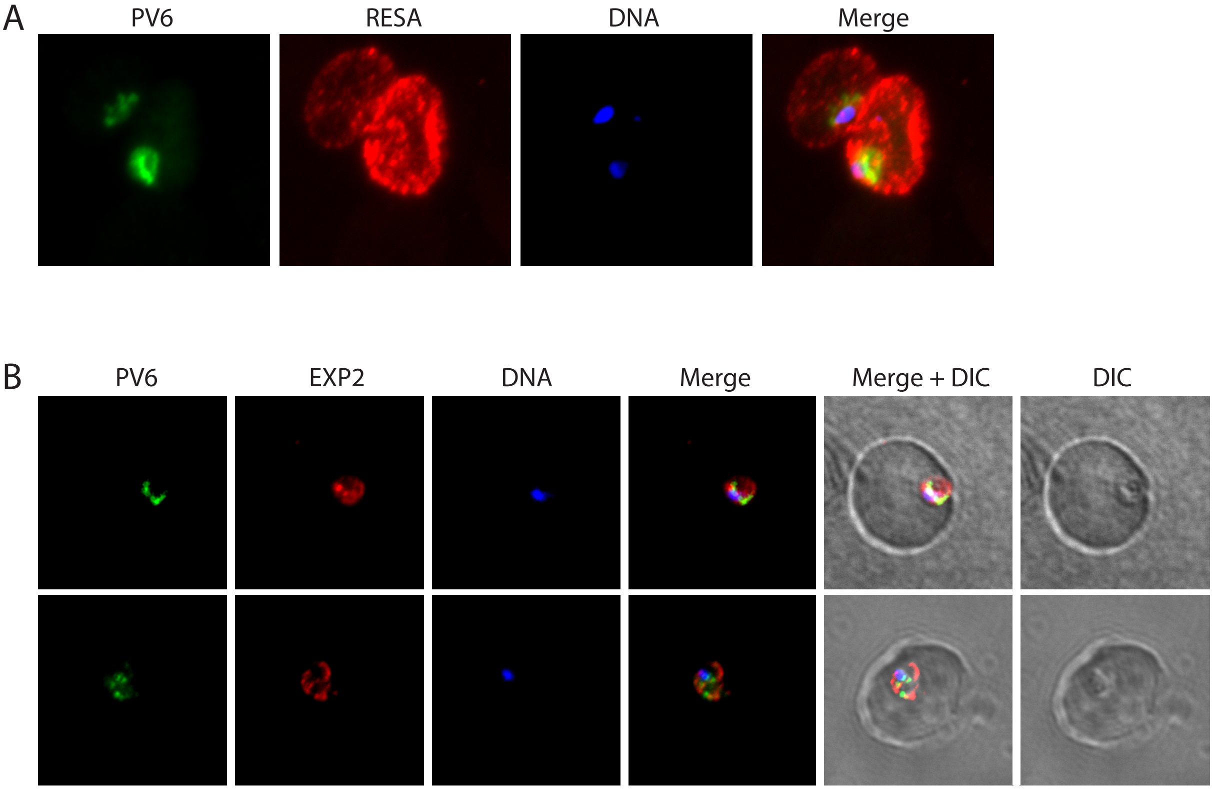

Supplementary Figure 2. Immunofluorescence assays of erythrocytes containing recently invaded parasites. A) Maximum projection view of infected erythrocytes shown in Figure 2B, bottom row, showing that both erythrocytes that display anti-RESA staining are infected. B) Co-staining of infected erythrocytes with anti-PV6 and anti-EXP2 antibodies after invasion. Note that although the proteins do not overlap to a large degree, they appear to be present in the same compartment.
