# Supplementary material for: Identification of a non-exported Plasmepsin V substrate that functions in the parasitophorous vacuole of malaria parasites
Source: mBio. 2023 Dec 11;15(1):e01223-23. doi: 10.1128/mbio.01223-23 (PMC10790765; doi:10.1128/mbio.01223-23)
Supplement: Figure S3 — Immunoblot SLO lysis of AMA1-HA-PV6 fusion and PV6-HA-PV6. [file mbio.01223-23-s0003.pdf]

Supplementary Figure 3

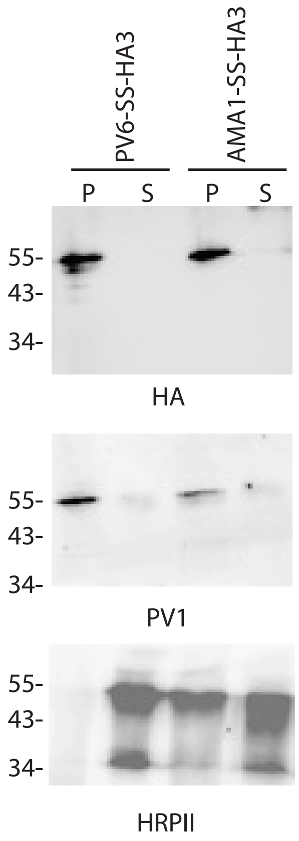

Supplementary Figure 3. Differential lysis of AMA1-SS-HA3-PV6 and PV6-HA3-PV6 infected erythrocytes with Streptolysin O (SLO). Infected erythrocytes (35-40 hours post-invasion) were treated with SLO, the erythrocytes were pelleted and the supernatant and pellet were collected. Samples were prepared for immunoblotting and probed with the indicated antibodies. Note the similarity in the release of AMA1-SS-HA3-PV6, PV6-HA3-PV6 and the parasitophorous vacuole marker PV1 in the samples.
