# Supplementary material for: Identification of a non-exported Plasmepsin V substrate that functions in the parasitophorous vacuole of malaria parasites
Source: mBio. 2023 Dec 11;15(1):e01223-23. doi: 10.1128/mbio.01223-23 (PMC10790765; doi:10.1128/mbio.01223-23)
Supplement: Figure S4 — Export of PV6 K64A mutant. [file mbio.01223-23-s0004.pdf]

Supplementary Figure 4

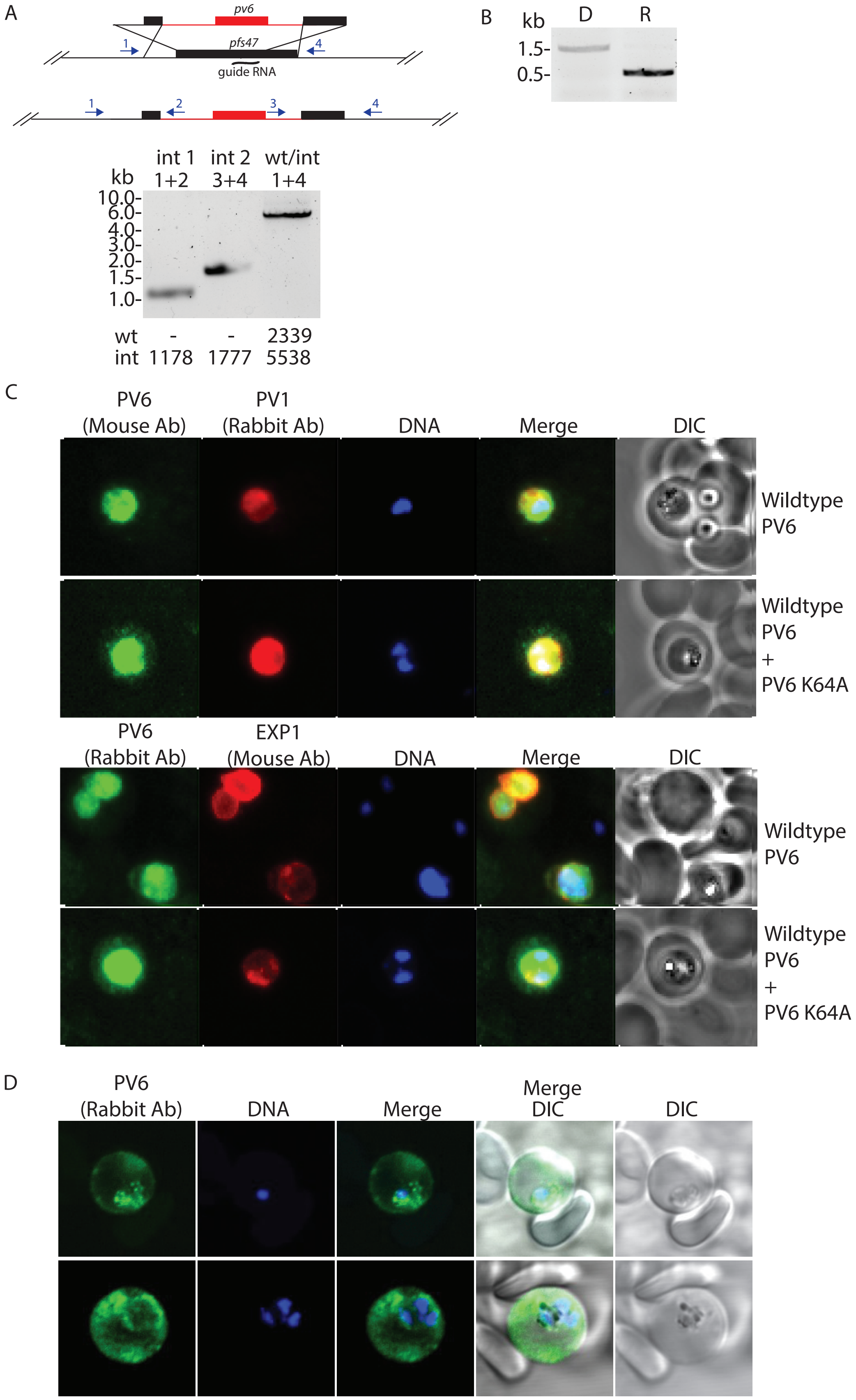

Supplementary Figure 4. Genetic modification of the *pfs47* locus and localization of PV6 K64A mutant protein. A) overview of the integration strategy using Cas9-mediated gene modification of the *pfs47* locus with the pv6 gene carrying the K64A mutation and PCR analysis of integration of parasites obtained. Indicated above are the primer pairs used in the PCR and underneath are the expected sizes of the PCR products (see also Primers Supp Table 1). B) Immunofluorescence assays of erythrocytes infected with wildtype parasites or parasites infected with parasites expressing an additional copy of PV6 containing a K64A mutation, as indicated. Cells were stained with antibodies against PV6 to visualize PV6 and either PV1 or EXP1 to visualize the PV or PVM, respectively. C) PCR analysis of the pv6 locus of parasites carrying the K64A mutant at the *pfs47* locus after treatment with DMSO (D) or rapamycin (R). For outline of PCR scheme, please see Hill *et al.* (1) D) Localization of PV6 K64A protein in parasites 35 hours post-invasion, where the native PV6 protein is missing. Synchronized parasites PfBLD529 were treated with rapamycin soon after invasion to remove the native gene, leaving the mutant protein as the only PV6 protein (see Figure 4D for proteins levels). PV6 was visualized by immunofluorescence microscopy using an anti-PV6 antibody. Note that the mutant protein is present in the erythrocyte and in the parasite itself, and to a much lesser extent in the PV, as compared to parasites that also express the native PV6.
