# Supplementary material for: Identification of a non-exported Plasmepsin V substrate that functions in the parasitophorous vacuole of malaria parasites
Source: mBio. 2023 Dec 11;15(1):e01223-23. doi: 10.1128/mbio.01223-23 (PMC10790765; doi:10.1128/mbio.01223-23)
Supplement: Figure S5 — Overview of exportomes. [file mbio.01223-23-s0005.pdf]

Supplementary Figure 5

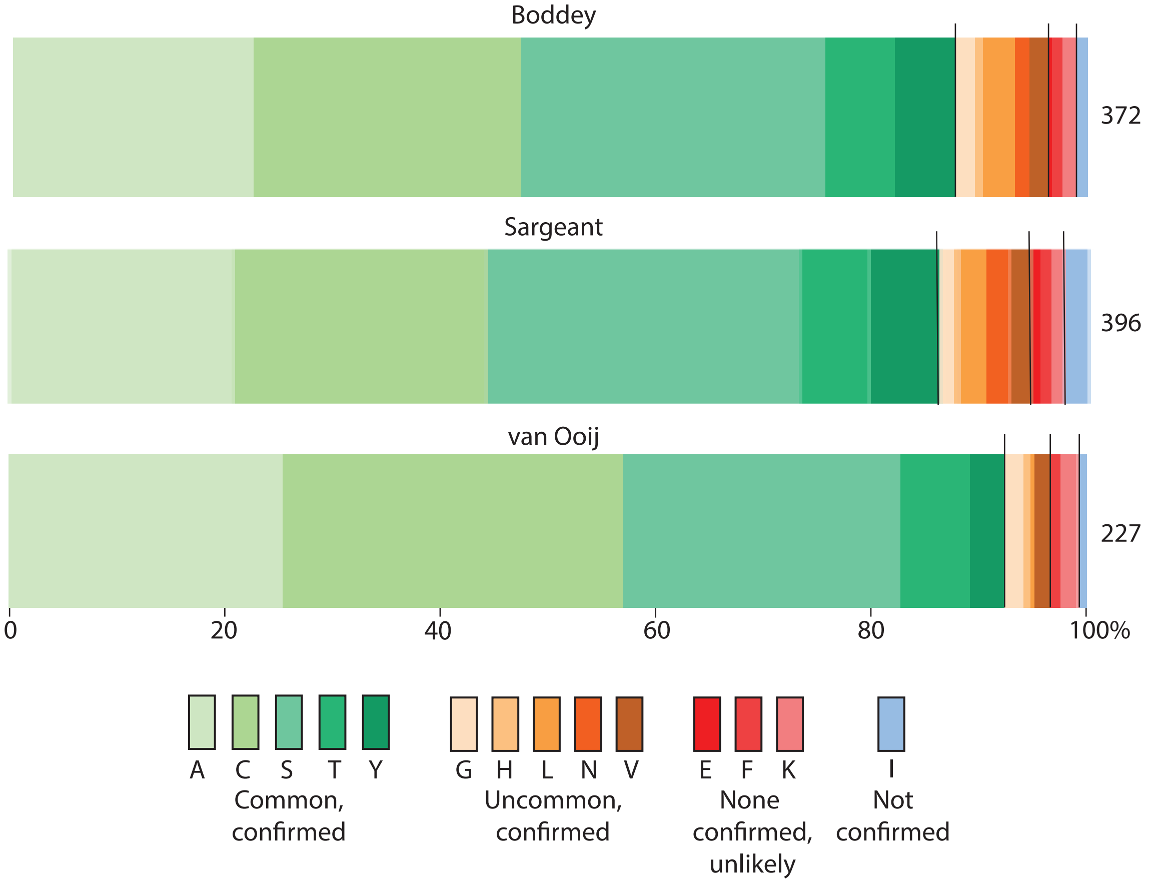

Supplementary Figure 5. Overview of prevalence of N-terminal amino acids in position 4 of the PEXEL in proteins identified in three different exportomes.
