# Supplementary material for: Identification of a non-exported Plasmepsin V substrate that functions in the parasitophorous vacuole of malaria parasites
Source: mBio. 2023 Dec 11;15(1):e01223-23. doi: 10.1128/mbio.01223-23 (PMC10790765; doi:10.1128/mbio.01223-23)
Supplement: Supplemental reference — Reference for supplemental material. [file mbio.01223-23-s0006.docx]

Supplementary material reference

1. Hill RJ, Ringel A, Knuepfer E, Moon RW, Blackman MJ, van Ooij C. 2016. Regulation and Essentiality of the StAR-related Lipid Transfer (START) Domain-containing Phospholipid Transfer Protein PFA0210c in Malaria Parasites. *J Biol Chem* 291:24280–24292.
